# Nuclear mechanobiology: a brief history and five unresolved questions

**DOI:** 10.1080/19491034.2026.2709894

**Published:** 2026-08-24

**Authors:** Jan Lammerding, Jorine Eeftens, Gregg G. Gundersen, Megan C. King, Tanmay P. Lele, Song Li, Gant Luxton, G. W., Ohad Medalia, Matthieu Piel, Pere Roca-Cusachs, Jörg Renkawitz, Kyle Roux, Daniel A. Starr, Amy R. Strom, Hawa Racine Thiam, Sara A. Wickström, Howard J. Worman

**Affiliations:** Weill Institute for Cell and Molecular Biology & Meinig School of Biomedical Engineering, Cornell University, Ithaca, NY, USA; Radboud Institute for Molecular Life Sciences, Radboud University, Nijmegen, The Netherlands; Department of Pathology and Cell Biology, Columbia University, New York, NY, USA; Department of Cell Biology, Yale School of Medicine, New Haven, CT, USA; Department of Biomedical Engineering, Texas A&M University, College Station, TX, USA; Department of Bioengineering, University of California Los Angeles, Los Angeles, CA, USA; Department of Molecular and Cellular Biology, University of California Davis; Davis, CA, USA; Department of Biochemistry, University of Zurich, Zurich, Switzerland; Cell Biology and Cancer, Paris, France; Institute for Bioengineering of Catalonia (IBEC), The Barcelona Institute of Science and Technology (BIST), Barcelona, Spain; Department of Biomedical Sciences, University of Barcelona, Barcelona, Spain; Biomedical Center, Walter Brendel Center of Experimental Medicine, Institute of Cardiovascular Physiology and Pathophysiology, Klinikum der Universität, Ludwig Maximilians Universität München, Munich, Germany; Center for Genetics and Rare Diseases Research, Sanford Research Institute, Sioux Falls, SD, USA; Department of Discovery Oncology, Genentech, South San Francisco, CA, USA; Department of Bioengineering and Department of Microbiology and Immunology, Sarafan ChEM-H Institute, Stanford University, Palo Alto, CA, USA; Biohub, San Francisco, CA, USA; Department of Cell and Tissue Dynamics, Max Planck Institute for Molecular Biomedicine, Münster, Germany; Department of Medicine, Columbia University, New York, NY, USA

The nucleus has long been recognized as the repository of the cell’s genomic information, providing the blueprints for assembling the cell’s structures and carrying out its functions. Over the past three decades, however, it has become abundantly clear that cellular structure and function are not only governed by the genome’s sequence, but also the physical structure and mechanical properties of the nucleus [1]. Furthermore, nuclear structure and mechanics are not fixed properties but respond dynamically to physical and biochemical cues from inside and outside the cell, thereby enabling cells to sense and adapt to their environment [2–4]. This intriguing interplay of how nuclear structure and mechanics modulate cellular functions, and how mechanical forces acting on the nucleus affect its structure and function is now commonly referred to as ‘nuclear mechanobiology.’

Some of the earliest work motivating the study of nuclear mechanobiology includes the recognition that compression of cartilage induces severe changes in nuclear shape and volume [5], and the elegant demonstration that extracellular forces are rapidly transmitted to the nucleus via the cytoskeleton, inducing intranuclear deformation [6]. This led to the hypothesis that the nucleus plays a central role in cellular mechanosensing. Since then, nuclear mechanobiology has grown into a rapidly expanding, interdisciplinary research area spanning cell and molecular biology, genomics, physics, engineering, and, increasingly, translational science and medicine. Subsequent studies have *(i)* shed light on how nuclear envelope proteins such as lamins and emerin, along with chromatin and intranuclear condensates, determine the physical properties of the nucleus [7–11], *(ii)* described characteristic changes in nuclear mechanics during differentiation and in response to the cellular physical environment [2,12], (*iii)* identified the molecular components involved in physically coupling the nucleus to the cytoskeleton, including the aptly named Linker of Nucleoskeleton and Cytoskeleton (LINC) complex [13–17], and *(iv)* demonstrated that the association and dissociation between the nuclear lamina and chromatin can drive chromatin remodeling [18–20]. These studies have laid the foundation for the discovery that the nucleus, and particularly the nuclear envelope, plays crucial roles in cellular mechanotransduction (i.e., the translation of mechanical cues into biochemical signals and changes in gene expression), both through structural reorganization and via biochemical signaling processes, thereby modulating cell differentiation, fate and survival [3,4,7,21–31].

Other studies have focused on how the physical properties of the cell nucleus, including its size, shape, and stiffness, modulate diverse cellular functions. Two areas of particular interest are the role of nuclear mechanobiology in *(i)* cell migration through confined spaces, e.g., during development, inflammation, and metastatic spreading of cancer cells, and *(ii)* diseases caused by mutations in genes encoding nuclear envelope proteins that affect skeletal and cardiac muscle and other mechanically active tissues. We now know that migration through tight spaces is limited by the deformability of the nucleus [32–38], that the nucleus can guide cells to find their path through complex 3D environments [39], and that the physical stress on the nucleus during confined migration can cause nuclear envelope rupture, DNA damage, altered chromatin organization, and changes in gene expression [40–48]. Mechanically induced nuclear damage has also emerged as an important disease driver in at least a subset of muscular dystrophies and cardiomyopathies caused by mutations in genes encoding nuclear envelope proteins such as lamins and emerin [7,49–53], with altered chromatin organization and transcription, in part resulting from perturbed tethering of chromatin to the nuclear periphery, likely serving as additional pathogenic mechanisms [54,55]. Intriguingly, reducing nuclear envelope rupture by disrupting the LINC complex has been shown to dramatically slow disease progression and extend survival in preclinical models of these diseases [50,56–59]. A clinical trial leveraging this approach, scheduled to begin soon [60], showcases the potential of nuclear mechanobiology impact on patient care.

As interest in nuclear mechanobiology continues to grow, new insights and applications emerge. Recent examples include the opening of nuclear pore complexes in response to nuclear membrane tension [24,25,61–63], substrate stiffness-induced changes in chromatin compaction and/or accessibility [64,65], the bidirectional interplay between mechanical stress, nuclear mechanics, and DNA damage repair pathways [66–70], and the role of nuclear mechanics in immune cell function [71–73]. We are excited to highlight and discuss the current knowledge in many of these research areas in a *Special Issue on Nuclear Mechanobiology*. The special issue includes reviews on nuclear mechanobiology confined migration [74], immune cell function [75], mechanotransduction at the nuclear envelope [76], the role of chromatin in nuclear mechanics [77], nuclear movement and migration [78], integration between nuclear mechanosensing and integrin adhesions [79], muscle aging [80], and novel tools to image nuclear structure by cryo-electron tomography [81] or study mechanical regulation of chromatin [82].

Nevertheless, even as the field of nuclear mechanobiology has made extensive progress, many open questions remain, and new questions continue to arise. To kick off this special issue, we reached out to top experts in nuclear mechanobiology and asked them to list what they consider the most pressing open research questions in the field today. More than 15 experts responded and agreed to serve as coauthors for this editorial. We then consolidated their input into five central open questions in nuclear mechanobiology. We recognize that such a list cannot be comprehensive and offers only a single snapshot of prevalent thoughts and ideas in the field, and we encourage readers to add and ponder additional questions. We hope that the following questions will inspire and motivate further research into the mechanobiology of the nucleus, so that we gain further insights into the fascinating interplay between nuclear structure, mechanics, and cellular functions. There is still so much to learn about mechanobiology and the nucleus!

## Five central open questions in nuclear mechanobiology

### The molecular basis of nuclear mechanics: which ultrastructural components and connections determine nuclear structure and stiffness, and how are they regulated?

Wide-ranging studies aimed at measuring nuclear biomechanics, using intact cells and isolated nuclei, have produced an extensive list of molecules that contribute to the mechanical properties of the nucleus. These contributors include various nuclear envelope proteins, such as lamins, emerin, LAP1, LAP2β, LBR, and LEMD2, as well as chromatin and histone modifications [7,9,19,83–91]. However, exactly how these molecules contribute to nuclear mechanics, including their ultrastructural organization and their interplay that gives rise to emergent properties, remains largely undetermined. For example, lamins and many other nuclear envelope proteins can directly interact with each other and with chromatin but can also form discrete networks and complexes [92–94]. We still lack a complete understanding of the composition, structure, and function of these protein complexes at the nuclear envelope, as many of the nuclear envelope proteins include intrinsically disordered domains that are challenging to resolve with current methods. Furthermore, elegant work in yeast showed that untethering chromatin from the nuclear envelope results in increased nuclear deformability [11]. However, the effect of interactions between chromatin and the nuclear lamina, nuclear pore complexes, and nucleoskeletal structures on nuclear mechanics in mammalian cells has proven difficult to untangle from the intrinsic mechanical properties of chromatin and its role in gene expression. New approaches to ‘visualize’ the nuclear lamina structure and chromatin organization, including both imaging techniques and molecular biology assays, will be needed to paint an accurate picture of nuclear ultrastructure and dynamics.

Even when focusing on individual molecules, numerous questions remain. For example, despite very similar structure and biochemical properties, A-type and B-type lamins differ substantially in their effect on nuclear deformability [85,92], but the molecular features responsible for this difference have yet to be determined. Additionally, many small nuclear envelope proteins such as emerin and short nesprin isoforms can be found both at the inner and other nuclear membrane; how their intracellular localization affects their contribution to nuclear mechanobiology is only beginning to emerge [26].

The nuclear interior poses many of its own questions. For example, how does biomolecular condensation influence nuclear mechanical properties and function, and conversely, how do mechanical forces on the nucleus—or generated within the nucleus during DNA replication, transcription, or loop extrusion—shape intranuclear architecture and condensate formation [95,96]? And how does macromolecular crowding, which can be affected by physical and biochemical cues [97,98], alter nuclear mechanics, function, and ultimately cell fate? Molecular crowding is emerging as an important mechanobiology variable [97,99,100], yet how mechanical forces alter local crowding, and how crowding modulates nuclear and cytoplasmic functions, remains largely unmapped.

Gaining further insights into the molecular and biophysical details governing nuclear mechanics will provide a deeper understanding of fundamental biological functions but might also reveal new clues into pathogenic processes and new therapeutic strategies.

### The adaptability of nuclear structure and mechanics: how do mechanical stimuli and other signals modulate nuclear mechanics?

Increasing evidence suggests that the mechanical properties of the nucleus are not static, but can change dynamically, for example, during confined migration [101], in response to acute force application [21], changes in substrate stiffness [2], or cell differentiation [2,12, 97, 98, 102, 103]. In some cases, the changes in nuclear mechanics can be attributed to altered lamin expression or chromatin organization [2,12,102,103], although the upstream mechanisms largely remain to be determined. In many other scenarios, it is still unclear how intra- and extranuclear cellular processes modulate nuclear organization and mechanical properties, for example, by altering macromolecular crowding, assembly of macromolecular complexes, and/or nuclear condensates. Similarly, it remains to be explored to what extent changes in nuclear mechanics result from direct mechanically induced protein conformational changes that affect protein–protein and protein-nucleic acid interactions, or downstream biochemical and signaling pathways. Additionally, the timescales required for such changes to occur or be reversed, and to what extent such changes can result in local variations in nuclear mechanics and function within the nucleus [104], are yet to be fully defined.

The simple fact that cells can rapidly ‘adjust’ their lamin levels to the substrate stiffness and viscoelasticity [2,31,105], yet different cell types retain the characteristic lamin levels of their tissue of origin in culture, even after numerous passages on stiff tissue culture plastic [2], indicates that there is still much to be learned about the dynamic regulation of nuclear mechanics. This includes cell cycle-dependent changes in nuclear mechanics, and the question whether nuclear mechanics are primarily determined during early post-mitotic events, e.g., nuclear envelope re-assembly and chromatin decondensation, or also later in the cell cycle. A fundamental question is how nuclear mechanical properties change over longer time periods, for example, during development or aging, and how such changes functionally contribute to both physiological and pathological processes.

Lastly, individual nuclei often display variability in chromatin compaction, nuclear body organization, and mechanical stiffness—regardless of whether studied *in vitro* or *in vivo*—but the origins and consequences of this heterogeneity are still not well understood. Answering this question might provide not only further clues into the factors that govern nuclear shape and function, but also how tissues and organs maintain robust function despite or because of this heterogeneity.

### Nuclear mechanosensing: how are mechanical forces on the nucleus translated into changes in gene expression and cellular signals?

The nucleus has long been proposed to serve as a cellular mechanosensor, but experimental data unequivocally documenting that forces or deformations applied to the nucleus are sufficient to activate cellular signaling pathways and expression of mechanoresponsive genes are still quite sparse. For example, whereas recent studies provided clear evidence that nuclear deformation triggers increased cell contractility, mediated by cPLA2 responding to changes in nuclear membrane tension [3,4,23,76,106], in many other cases it remains unclear whether observed changes in chromatin organization or gene expression result from mechanosensing events within the nucleus or downstream of mechanotransduction processes at the plasma membrane or in the cytoplasm. Thus, it largely remains to be resolved if, how, and to what extent, forces transmitted to the nucleus are transduced into changes in chromatin organization, gene expression, and epigenetic modifications, and which molecules and molecular pathways are involved in the associated processes. For example, are physical forces and deformations sufficient to induce chromatin opening, conformational changes in nuclear proteins, or alter intermolecular interactions (e.g., protein–protein binding, condensate formation, or protein-nucleic acid binding) under physiological conditions, thereby modulating transcriptional regulation? Or do the observed transcriptional changes require intermediate messengers and effectors, such as specific transcription factors, histone modifiers, or kinases, which could provide a mechanism to modulate the expression of specific genes? In the latter case it remains to be resolved how these modifiers are activated by mechanical stimuli, how such stimuli can achieve specificity, and whether these mechanoresponses employ mechanisms distinct from those associated with biochemical stimuli? Although recent studies support various putative mechanisms of nuclear mechanosensing [1,2,18,27,30,47,107], further work is needed to fully elucidate the underlying processes and identify the associated molecular players. It is also unclear whether nuclear tension, compression, and other deformations are sensed through the same or distinct mechanisms, and how such mechanical stimuli are integrated with cytoplasmic mechanotransduction pathways and input from other signals, such as biochemical stimulation, to inform cellular decision-making.

One illustrative example is the modulation of nucleo-cytoplasmic trafficking by nuclear membrane tension-mediated changes in the diameter of the nuclear pore complexes [24,25]. Based on cryo-electron tomography imaging, the nuclear pore complex central channel increases from 43 nm to 57 nm when comparing isolated, tension-free nuclear envelope samples with nuclear pores *in situ* in fixed HeLa cells [108]. Smaller, but still significant changes in the nuclear pore diameter can also be detected in response to osmotic shock [61], or when cells are grown on soft versus stiff substrates [24,25], the latter of which cause nuclear flattening and increase membrane tension, and these changes are associated with increased nuclear transport. However, since numerous other factors also regulate nuclear transport of specific cargoes, including their posttranslational modifications, association with importins and exportins, the availability of co-transporters such as G-actin, retention by cytoplasmic or nuclear binding partners, and the intracellular Ran-GTP gradient [109,110], which can all be affected by the cell’s physical environment, parsing out the contributions from nuclear membrane tension and changes in nuclear pore complex diameter versus other factors remains extremely challenging. Besides the mechanism of nuclear mechanosensing, other open questions revolve around the mechanical memory. This memory includes altered nuclear structure and cellular behavior that persists after the initial mechanical stimulus has been removed, along with how such mechanical memory is implemented, maintained, and, in many cases, eventually erased.

Answering these questions is made more challenging by the growing realization that many methods to disrupt nucleo-cytoskeletal force transmission, such as depletion of LINC complex components or LINC complex disruption using dominant-negative constructs, also affect nuclear and cytoskeletal organization, along with various signaling pathways, such as Wnt signaling [111–117]. For this reason, it difficult to unambiguously distinguish between true nuclear mechanotransduction and other pathways.

### Force transmission across the nuclear envelope: do we understand the structure and function of the LINC complex correctly, and how is the mechanical coupling between the cytoskeleton and nucleus regulated?

The LINC complex is comprised of KASH domain proteins, called nesprins in mammals, in the outer nuclear membrane that connect to SUN domain protein trimers embedded in the inner nuclear membrane [13]. Since nesprins simultaneously interact with various cytoskeletal structures in the cytoplasm, and SUN proteins with lamins, nuclear pore complexes, and chromatin at the nuclear interior, this connection enables a physical link across the nuclear envelope. Consequently, over the past 20 years, the LINC complex has emerged as a central structure in mediating force transmission between the nucleus and cytoskeleton [13,114,118]. However, just as focal adhesions, adherens junctions, and desmosomes that mediate force transmission at the cell surface are not static mechanical connections but instead operate like a clutch that is responsive to inputs from inside and outside the cell, the interaction of nesprins and SUN proteins, along with their interaction with other binding partners, must be dynamically regulated to produce specific cellular outcomes. For example, cytoskeletal forces result in nuclear movement in some cases, while the nucleus can be anchored in place and resist movement in other cases, and in specific scenarios, such as meiosis, move chromosomes within the nucleus. Although recent imaging, modeling, and functional studies have shed some light on the interactions between nesprins and SUN proteins at the nuclear envelope [119–127], how this interaction is regulated, including the identity of relevant cellular signals and other molecules involved in the regulation [128], is still largely unknown. This is in part because the large size, flexible structure, and numerous isoforms of nesprins present substantial challenges in mapping their structure and function at the nuclear envelope.

In the case of focal adhesions, more than five decades of research have produced a nanoscale understanding of the architecture of the over 300 proteins that make up focal adhesions [129–133] and their role in mechanical force transmission and mechanosensing. Yet much of this information is still lacking for the LINC complex and its associated proteins, including its 3D topological architecture on the surface of the nucleus and the structure, function, and composition of the macromolecular complexes at the inner nuclear membrane involved in transmitting forces to the nuclear interior. Notably, although most illustrations of the LINC complex still depict nesprins as long radial elements spanning large distances from the nucleus, recent studies suggest a different organization. Nesprins contain numerous spectrin-like repeats, which are commonly associated with membrane-proximal scaffolds [134,135], and likely form highly connected networks at the surface of the nuclear envelope, rather than projecting radially from the nucleus as isolated linkers [136–141]. The traditional view of the LINC complex is further complicated by the fact that, although nesprins are typically considered outer nuclear membrane proteins, shorter nesprin isoforms localize to the inner nuclear membrane, where they interact with lamins and chromatin [136,138,142].

Besides the LINC complex, other nuclear envelope structures contribute to nucleo-cytoskeletal coupling and force transmission, including nuclear pore complexes [143,144], emerin [21,37,145,146], and NEMP1 [147], but the relative contributions of these structures to overall force transmission, and the specific context in which one structure might be favored over another, remain incompletely understood. In addition, it is still unclear how nuclear membrane tension, which can modulate transit through nuclear pore complexes, opening of stretch sensitive ion channels, and association between cPLA2 and nuclear membrane [3,4,23–25,27,76], is transmitted to and propagated through the nuclear envelope, and what roles the LINC complex, lamins, and other nuclear envelope proteins play in this context. Answering these questions will likely require advances in imaging of the nuclear envelope at nanoscale resolution, along with innovative experimental approaches to delineate the contribution of specific nuclear envelope proteins.

### Nuclear mechanobiology meets the clinic: how well do 2D in vitro studies represent physiological and pathological 3D in vivo conditions, and how can we use insights from tissue culture studies to develop deeper insights and new therapies for human diseases?

Changes in nuclear organization and mechanics associated with aging and pathological conditions, such as during cancer progression, cardiovascular disease, and muscular dystrophies [148–150], have long provided a strong motivation to study nuclear mechanobiology. Nonetheless, most studies on nuclear mechanics and nuclear mechanobiology are still being conducted on cultured cells or even isolated nuclei, owing to the relative ease of experiments compared to *in vivo* studies, the increased control over experimental conditions, and the easier interpretation of results given fewer confounding factors. However, both cellular and nuclear morphology and function differ substantially between 2D and 3D conditions, and between culture on rigid substrates such as glass or polystyrene cell culture surfaces (Young’s modulus: 3–75 GPa) versus materials with physiological stiffness (0.1–100 kPa, i.e., almost 10^6^-times softer). For example, while many cell types cultured on rigid 2D substrates exhibit extensive cell spreading resulting in flattened, disc-shaped nuclei often only a few micrometers in height, the same cells cultured in 3D environments or imaged *in vivo* have substantially thicker, more spherical, and larger nuclei, which is likely to affect the forces acting on the nucleus, along with nuclear crowding, mechanics, and intranuclear organization [41,151–154]. Thus, it will be crucial to determine how well 2D tissue culture models represent cellular and nuclear structure, architecture, and function *in vivo*, and how well our understanding of nuclear mechanobiology relates to the condition of cells in tissues, organs, and whole organisms. The increasing use of 3D culture systems, organoids, and *in situ* systems that better reflect the natural environment of cells, plus the use of model systems that enable intravital imaging and functional measurements, such as zebrafish, *Drosophila*, and *C. elegans*, provide critical complements to the cell culture systems.

Lastly, beyond advancing our fundamental understanding of key biological processes, mechanobiology is increasingly beginning to inform clinical applications, including new therapeutic strategies and diagnostic assays [155]. Clinical applications in nuclear mechanobiology are slowly starting to emerge, with the first clinical trial using LINC complex disruption to treat dilated cardiomyopathy caused by pathogenic lamin A/C gene mutations recently approved by the United States Food and Drug Administration [60]. Other recent studies linked nuclear envelope mechanical stability, mediated by lamin A/C and SUN2, to the therapeutic mechanism of paclitaxel [156,157], which is commonly used in chemotherapy. Thus, a central question moving forward will be how to harness insights from nuclear mechanobiology for clinical application. Answering this challenge could inspire new diagnostic and therapeutic breakthroughs across a broad spectrum of human diseases, spanning development, cancer, musculoskeletal disease, immune cell dysfunction, and aging. We are excited to follow the payoffs of nuclear mechanobiology over the coming decades!

## Data Availability

Data sharing not applicable – no new data generated.

## References

[1] Kalukula Y, Stephens AD, Lammerding J, et al. Mechanics and functional consequences of nuclear deformations. Nat Rev Mol Cell Biol. 2022;23(9):583–13. doi: 10.1038/s41580-022-00480-z 35513718 PMC9902167

[2] Swift J, Ivanovska IL, Buxboim A, et al. Nuclear lamin-A scales with tissue stiffness and enhances matrix-directed differentiation. Science. 2013;341(6149):1240104. doi: 10.1126/science.1240104 23990565 PMC3976548

[3] Lomakin AJ, Cattin CJ, Cuvelier D, et al. The nucleus acts as a ruler tailoring cell responses to spatial constraints. Science. 2020;370(6514):eaba2894. doi: 10.1126/science.aba2894 33060332 PMC8059074

[4] Venturini V, Pezzano F, Català Castro F, et al. The nucleus measures shape changes for cellular proprioception to control dynamic cell behavior. Science. 2020;370(6514):eaba2644. doi: 10.1126/science.aba2644 33060331

[5] Guilak F. Compression-induced changes in the shape and volume of the chondrocyte nucleus. J Biomech. 1995;28(12):1529–1541. doi: 10.1016/0021-9290(95)00100-x 8666592

[6] Maniotis AJ, Chen CS, Ingber DE. Demonstration of mechanical connections between integrins, cytoskeletal filaments, and nucleoplasm that stabilize nuclear structure. Proc Natl Acad Sci USA. 1997;94(3):849–854. doi: 10.1073/pnas.94.3.849 9023345 PMC19602

[7] Lammerding J, Schulze PC, Takahashi T, et al. Lamin A/C deficiency causes defective nuclear mechanics and mechanotransduction. J Clin Invest. 2004;113(3):370–378. doi: 10.1172/JCI200419670 14755334 PMC324542

[8] Dahl KN, Kahn SM, Wilson KL, et al. The nuclear envelope lamina network has elasticity and a compressibility limit suggestive of a molecular shock absorber. J Cell Sci. 2004;117(20):4779–4786. doi: 10.1242/jcs.01357 15331638

[9] Stephens AD, Banigan EJ, Adam SA, et al. Chromatin and lamin a determine two different mechanical response regimes of the cell nucleus. MBoC. 2017;28(14):1984–1996. doi: 10.1091/mbc.e16-09-0653 28057760 PMC5541848

[10] Strom AR, Kim Y, Zhao H, et al. Condensate interfacial forces reposition DNA loci and probe chromatin viscoelasticity. Cell. 2024. doi: 10.1016/j.cell.2024.07.034 39168125

[11] Schreiner SM, Koo PK, Zhao Y, et al. The tethering of chromatin to the nuclear envelope supports nuclear mechanics. Nat Commun. 2015;6():7159. doi: 10.1038/ncomms8159. 26074052 PMC4490570

[12] Pajerowski JD, Dahl KN, Zhong FL, et al. Physical plasticity of the nucleus in stem cell differentiation. Proc Natl Acad Sci USA. 2007;104(40):15619–15624. doi: 10.1073/pnas.0702576104 17893336 PMC2000408

[13] Crisp M, Liu Q, Roux K, et al. Coupling of the nucleus and cytoplasm: role of the LINC complex. J Cell Biol. 2006;172(1):41–53. doi: 10.1083/jcb.200509124 16380439 PMC2063530

[14] Haque F, Lloyd DJ, Smallwood DT, et al. SUN1 interacts with nuclear lamin a and cytoplasmic nesprins to provide a physical connection between the nuclear lamina and the cytoskeleton. Mol Cell Biol. 2006;26(10):3738–3751. doi: 10.1128/MCB.26.10.3738-3751.2006 16648470 PMC1488999

[15] Starr DA, Han M. Role of ANC-1 in tethering nuclei to the actin cytoskeleton. Science. 2002;298(5592):406–409. doi: 10.1126/science.1075119 12169658

[16] Starr DA, Fridolfsson HN. Interactions between nuclei and the cytoskeleton are mediated by SUN-KASH nuclear-envelope bridges. Annu Rev Cell Dev Biol. 2010;26(2010):421–444. doi: 10.1146/annurev-cellbio-100109-104037 20507227 PMC4053175

[17] Luxton GWG, Gomes ER, Folker ES, et al. Linear arrays of nuclear envelope proteins harness retrograde actin flow for nuclear movement. Science. 2010;329(5994):956–959. doi: 10.1126/science.1189072 20724637 PMC3938394

[18] Song Y, Soto J, Chen B, et al. Transient nuclear deformation primes epigenetic state and promotes cell reprogramming. Nat Mater. 2022;21(10):1191–1199. doi: 10.1038/s41563-022-01312-3 35927431 PMC9529815

[19] Lewis R, Sinigiani V, Maziak N, et al. LBR and LAP2 mediate heterochromatin tethering to the nuclear periphery to preserve genome homeostasis. Nat Cell Biol. 2026;28(3):536–552. doi: 10.1038/s41556-025-01822-7 41735607 PMC12992122

[20] Buchwalter A, Kaneshiro JM, Hetzer MW. Coaching from the sidelines: the nuclear periphery in genome regulation. Nat Rev Genet. 2019;20(1):39–50. doi: 10.1038/s41576-018-0063-5 30356165 PMC6355253

[21] Guilluy C, Osborne LD, Van Landeghem L, et al. Isolated nuclei adapt to force and reveal a mechanotransduction pathway in the nucleus. Nat Cell Biol. 2014;16(4):376–381. doi: 10.1038/ncb2927 24609268 PMC4085695

[22] Carley E, Stewart RM, Zieman A, et al. The LINC complex transmits integrin-dependent tension to the nuclear lamina and represses epidermal differentiation. Elife. 2021;10:e58541. doi: 10.7554/eLife.58541 33779546 PMC8051949

[23] Enyedi B, Jelcic M, Niethammer P. The Cell nucleus serves as a mechanotransducer of tissue damage-induced inflammation. Cell. 2016;165(5):1160–1170. doi: 10.1016/j.cell.2016.04.016 27203112 PMC4875569

[24] Elosegui-Artola A, Andreu I, Beedle AEM, et al. Force triggers YAP nuclear entry by regulating transport across nuclear pores. Cell. 2017;171(6):1397–1410.e14. doi: 10.1016/j.cell.2017.10.008 29107331

[25] Andreu I, Granero-Moya I, Chahare NR, et al. mechanical force application to the nucleus regulates nucleocytoplasmic transport. Nat Cell Biol. 2022;24(6):896–905. doi: 10.1038/s41556-022-00927-7 35681009 PMC7614780

[26] Le HQ, Ghatak S, Yeung CYC, et al. mechanical regulation of transcription controls polycomb-mediated gene silencing during lineage commitment. Nat Cell Biol. 2016;18(8):864–875. doi: 10.1038/ncb3387 27398909

[27] Nava MM, Miroshnikova YA, Biggs LC, et al. Heterochromatin-driven nuclear softening protects the genome against mechanical stress-induced damage. Cell. 2020;181(4):800–817.e22. doi: 10.1016/j.cell.2020.03.052 32302590 PMC7237863

[28] Iyer KV, Pulford S, Mogilner A, et al. mechanical activation of cells induces chromatin remodeling preceding MKL nuclear transport. Biophys J. 2012;103(7):1416–1428. doi: 10.1016/j.bpj.2012.08.041 23062334 PMC3471483

[29] Alisafaei F, Jokhun DS, Shivashankar GV, et al. Regulation of nuclear architecture, mechanics, and nucleocytoplasmic shuttling of epigenetic factors by cell geometric constraints. Proc Natl Acad Sci USA. 2019;116(27):13200–13209. doi: 10.1073/pnas.1902035116 31209017 PMC6613080

[30] Tajik A, Zhang Y, Wei F, et al. Transcription upregulation via force-induced direct stretching of chromatin. Nat Mater. 2016;15(12):1287–1296. doi: 10.1038/nmat4729 27548707 PMC5121013

[31] Wu Y, Song Y, Soto J, et al. Viscoelastic extracellular matrix enhances epigenetic remodeling and cellular plasticity. Nat Commun. 2025;16(1):4054. doi: 10.1038/s41467-025-59190-7. 40307238 PMC12043949

[32] Wolf K, Te Lindert M, Krause M, et al. Physical limits of cell migration: control by ECM space and nuclear deformation and tuning by proteolysis and traction force. J Cell Bio. 2013;201(7):1069–1084. doi: 10.1083/jcb.201210152 23798731 PMC3691458

[33] Harada T, Swift J, Irianto J, et al. Nuclear lamin stiffness is a barrier to 3D migration, but softness can limit survival. J Cell Bio. 2014;204(5):669–682. doi: 10.1083/jcb.201308029 24567359 PMC3941057

[34] Rowat AC, Jaalouk DE, Zwerger M, et al. Nuclear envelope composition determines the ability of neutrophil-type cells to passage through micron-scale constrictions. J Biol Chem. 2013;288(12):8610–8618. doi: 10.1074/jbc.M112.441535 23355469 PMC3605679

[35] Davidson PM, Denais C, Bakshi MC, et al. Nuclear deformability constitutes a rate-limiting step during cell migration in 3-D environments. Cell Mol Bioeng. 2014;7(3):293–306. doi: 10.1007/s12195-014-0342-y 25436017 PMC4243304

[36] Thiam HR, Vargas P, Carpi N, et al. Perinuclear Arp2/3-driven actin polymerization enables nuclear deformation to facilitate cell migration through complex environments. Nat Commun. 2016;7(1):10997. doi: 10.1038/ncomms10997 26975831 PMC4796365

[37] Liddane AG, McNamara CA, Campbell MC, et al. Defects in emerin-nucleoskeleton binding disrupt nuclear structure and promote breast cancer Cell motility and metastasis. Mol Cancer Res. 2021;19(7):1196–1207. doi: 10.1158/1541-7786.MCR-20-0413 33771882 PMC8254762

[38] Lautscham LA, Kämmerer C, Lange JR, et al. Migration in confined 3D environments is determined by a combination of adhesiveness, nuclear volume, contractility, and Cell stiffness. Biophys J. 2015;109(5):900–913. doi: 10.1016/j.bpj.2015.07.025 26331248 PMC4564685

[39] Renkawitz J, Kopf A, Stopp J, et al. Nuclear positioning facilitates amoeboid migration along the path of least resistance. Nature. 2019;568(7753):546–550. doi: 10.1038/s41586-019-1087-5 30944468 PMC7217284

[40] Raab M, Gentili M, De Belly H, et al. ESCRT III repairs nuclear envelope ruptures during cell migration to limit DNA damage and cell death. Science. 2016;352(6283):359–362. doi: 10.1126/science.aad7611 27013426

[41] Denais CM, Gilbert RM, Isermann P, et al. Nuclear envelope rupture and repair during cancer cell migration. Science. 2016;352(6283):353–358. doi: 10.1126/science.aad7297 27013428 PMC4833568

[42] Irianto J, Xia Y, Pfeifer CR, et al. DNA damage follows repair factor depletion and portends genome variation in cancer cells after pore migration. Curr Biol. 2017;27(2):210–223. doi: 10.1016/j.cub.2016.11.049 27989676 PMC5262636

[43] Shah P, Hobson CM, Cheng S, et al. Nuclear deformation causes DNA damage by increasing replication stress. Curr Biol. 2021;31(4):753–765.e6. doi: 10.1016/j.cub.2020.11.037. 33326770 PMC7904640

[44] Hsia CR, McAllister J, Hasan O, et al. Confined migration induces heterochromatin formation and alters chromatin accessibility. iScience. 2022;25(9):104978. doi: 10.1016/j.isci.2022.104978 36117991 PMC9474860

[45] Jacobson EC, Perry JK, Long DS, et al. Migration through a small pore disrupts inactive chromatin organization in neutrophil-like cells. BMC Biol. 2018;16(1):142. doi: 10.1186/s12915-018-0608-2 30477489 PMC6257957

[46] Golloshi R, Playter C, Freeman TF, et al. Constricted migration is associated with stable 3D genome structure differences in cancer cells. EMBO Rep. 2022;23(10):e52149. doi: 10.15252/embr.202052149 35969179 PMC9535800

[47] Zhao JZ, Xia J, Brangwynne CP. Chromatin compaction during confined cell migration induces and reshapes nuclear condensates. Nat Commun. 2024;15(1):9964. doi: 10.1038/s41467-024-54120-5 39557835 PMC11574006

[48] Bastianello G, Kidiyoor GR, Lowndes C, et al. mechanical stress during confined migration causes aberrant mitoses and c-MYC amplification. Proc Natl Acad Sci USA. 2024;121(29):e2404551121. doi: 10.1073/pnas.2404551121 38990945 PMC11260125

[49] Cho S, Vashisth M, Abbas A, et al. Mechanosensing by the lamina protects against nuclear rupture, DNA damage, and cell cycle arrest. Dev Cell. 2019;49(6):920–935.e5. doi: 10.1016/j.devcel.2019.04.020 31105008 PMC6581604

[50] Earle AJ, Kirby TJ, Fedorchak GR, et al. Mutant lamins cause nuclear envelope rupture and DNA damage in skeletal muscle cells. Nat Mater. 2020;19(4):464–473. doi: 10.1038/s41563-019-0563-5 31844279 PMC7102937

[51] Zwerger M, Jaalouk DE, Lombardi ML, et al. Myopathic lamin mutations impair nuclear stability in cells and tissue and disrupt nucleo-cytoskeletal coupling. Hum Mol Genet. 2013;22(12):2335–2349. doi: 10.1093/hmg/ddt079. 23427149 PMC3658163

[52] En A, Bogireddi H, Thomas B, et al. Pervasive nuclear envelope ruptures precede ECM signaling and disease onset without activating cGAS-STING in lamin-cardiomyopathy mice. Cell Rep. 2024;43(6):114284. doi: 10.1016/j.celrep.2024.114284 38814785 PMC11290591

[53] Sikder K, Phillips E, Zhong Z, et al. Perinuclear damage from nuclear envelope deterioration elicits stress responses that contribute to LMNA cardiomyopathy. Sci Adv. 2024;10(19):eadh0798. doi: 10.1126/sciadv.adh0798 38718107 PMC11078192

[54] Mattout A, Pike BL, Towbin BD, et al. An EDMD mutation in C. elegans lamin blocks muscle-specific gene relocation and compromises muscle integrity. Curr Biol. 2011;21(19):1603–1614. doi: 10.1016/j.cub.2011.08.030. 21962710

[55] Cheedipudi SM, Matkovich SJ, Coarfa C, et al. Genomic reorganization of lamin-associated domains in cardiac myocytes is associated with differential gene expression and DNA methylation in human dilated cardiomyopathy. Circ Res. 2019;124(8):1198–1213. doi: 10.1161/CIRCRESAHA.118.314177 30739589 PMC6459729

[56] Chai RJ, Werner H, Li PY, et al. Disrupting the LINC complex by AAV mediated gene transduction prevents progression of lamin induced cardiomyopathy. Nat Commun. 2021;12(1):4722. doi: 10.1038/s41467-021-24849-4 34354059 PMC8342462

[57] Leong EL, Khaing NT, Cadot B, et al. Nesprin-1 LINC complexes recruit microtubule cytoskeleton proteins and drive pathology in *Lmna* -mutant striated muscle. Hum Mol Genet. 2023;32(2):177–191. doi: 10.1093/hmg/ddac179 35925868 PMC9840208

[58] Amiad Pavlov D, Heffler J, Suay-Corredera C, et al. Microtubule forces drive nuclear damage in LMNA cardiomyopathy. Nat Cardiovasc Res. 2025;4(11):1501–1520. doi: 10.1038/s44161-025-00727-w 41073815 PMC12611788

[59] Tan CY, Chan PS, Tan H, et al. Systematic in vivo candidate evaluation uncovers therapeutic targets for LMNA dilated cardiomyopathy and risk of lamin a toxicity. J Transl Med. 2023;21(1):690. doi: 10.1186/s12967-023-04542-4 37840136 PMC10577912

[60] Ng SR, Wang A, Tukijan F, et al. Abstract 4359043: NVC-001 – an AAV gene therapy functional cure for LMNA dilated cardiomyopathy. Circulation. 2025;152(Suppl_3):A4359043. doi: 10.1161/circ.152.suppl_3.4359043

[61] Zimmerli CE, Allegretti M, Rantos V, et al. Nuclear pores dilate and constrict in cellulo. Science. 2021;374(6573):eabd9776. doi: 10.1126/science.abd9776 34762489

[62] Lusk CP, Morgan KJ, King MC. Nuclear mechanics as a determinant of nuclear pore complex plasticity. Nat Cell Biol. 2025;27(10):1622–1631. doi: 10.1038/s41556-025-01768-w 40973737

[63] Morgan KJ, Carley E, Coyne AN, et al. Visualizing nuclear pore complex plasticity with pan-expansion microscopy. J Cell Biol. 2025;224(9):e202409120. doi: 10.1083/jcb.202409120 40504117 PMC12162248

[64] Heo SJ, Thakur S, Chen X, et al. Aberrant chromatin reorganization in cells from diseased fibrous connective tissue in response to altered chemomechanical cues. Nat Biomed Eng. 2023;7(2):177–191. doi: 10.1038/s41551-022-00910-5 35996026 PMC10053755

[65] Song Y, Soto J, Wong SY, et al. Biphasic regulation of epigenetic state by matrix stiffness during cell reprogramming. Sci Adv. 2024;10(7):eadk0639. doi: 10.1126/sciadv.adk0639 38354231 PMC10866547

[66] Kidiyoor GR, Li Q, Bastianello G, et al. ATR is essential for preservation of cell mechanics and nuclear integrity during interstitial migration. Nat Commun. 2020;11(1):4828. doi: 10.1038/s41467-020-18580-9 32973141 PMC7518249

[67] Bastianello G, Porcella G, Beznoussenko GV, et al. Cell stretching activates an ATM mechano-transduction pathway that remodels cytoskeleton and chromatin. Cell Rep. 2023;42(12):113555. doi: 10.1016/j.celrep.2023.113555 38088930

[68] Shah P, McGuigan CW, Cheng S, et al. ATM modulates nuclear mechanics by regulating lamin a levels. Front Cell Dev Biol. 2022;10:875132. doi: 10.3389/fcell.2022.875132 35721517 PMC9198445

[69] Kovacs MT, Vallette M, Wiertsema P, et al. DNA damage induces nuclear envelope rupture through ATR-mediated phosphorylation of lamin A/C. Mol Cell. 2023;83(20):3659–3668.e10. doi: 10.1016/j.molcel.2023.09.023 37832547 PMC10597398

[70] Joo YK, Black EM, Trier I, et al. ATR promotes clearance of damaged DNA and damaged cells by rupturing micronuclei. Mol Cell. 2023;83(20):3642–3658.e4. doi: 10.1016/j.molcel.2023.09.003 37788673 PMC10599252

[71] Thiam HR, Wong SL, Qiu R, et al. Netosis proceeds by cytoskeleton and endomembrane disassembly and PAD4-mediated chromatin decondensation and nuclear envelope rupture. Proc Natl Acad Sci USA. 2020;117(13):7326–7337. doi: 10.1073/pnas.1909546117 32170015 PMC7132277

[72] González-Granado JM, Silvestre-Roig C, Rocha-Perugini V, et al. Nuclear envelope lamin-A couples actin dynamics with immunological synapse architecture and T Cell activation. Sci Signal. 2014;7(322). doi: 10.1126/scisignal.2004872 PMC4337980 24757177

[73] Alraies Z, Rivera CA, Delgado MG, et al. Cell shape sensing licenses dendritic cells for homeostatic migration to lymph nodes. Nat Immunol. 2024;25(7):1193–1206. doi: 10.1038/s41590-024-01856-3 38834865 PMC11224020

[74] Patel H, Kaur S, Dickinson RB, et al. Nuclear mechanobiology in confined cell migration. Nucleus. 2026;17(1):2620879. doi: 10.1080/19491034.2026.2620879 41610884 PMC12867410

[75] Renkawitz J, Yesin A, Kroll J, et al. Nuclear mechanobiology rules immune cells’ functions: from differentiation to cell trafficking and pathogen killing. Nucleus. 2026;17(1):2590843. doi: 10.1080/19491034.2025.2590843 41495604 PMC12785203

[76] Rajan SG, Roca-Cusachs P, Niethammer P. Mechanotransduction by nuclear envelope tension. Nucleus. 2026;17(1):2600901. doi: 10.1080/19491034.2025.2600901 41402996 PMC12716060

[77] Rodenburg WS, Strom AR, Eeftens JM. Heterogeneity as a feature: unraveling chromatin’s role in nuclear mechanics. Nucleus. 2025;16(1):2545037. doi: 10.1080/19491034.2025.2545037 40838526 PMC12372515

[78] Gümüşderelioğlu S, Gant Luxton GW, Starr DA. Nuclei on the move: LINC complex-dependent and -independent mechanisms of nuclear migration in development. Nucleus. 2026;17(1):2679812. doi: 10.1080/19491034.2026.2679812 42218788 PMC13224763

[79] Sandria S, King MC. Integration of nuclear mechanosensing with integrin-extracellular matrix adhesions. Nucleus. 2026;17(1):2677308. doi: 10.1080/19491034.2026.2677308 42209418 PMC13220845

[80] Esen O, Battey E, Stroud MJ, et al. The nucleus as a mechanobiological hub in muscle aging. Nucleus. 2026;17(1):2688584. doi: 10.1080/19491034.2026.2688584 42316962 PMC13285602

[81] Kechagia Z, Medalia O. High-resolution nuclear cell biology by cryo-electron tomography. Nucleus. 2026;17(1):2675754. doi: 10.1080/19491034.2026.2675754 42226577 PMC13232881

[82] Soto J, Origer N, Wu Y, et al. Nuclear mechanotransduction: tools for mechanical perturbation and chromatin characterization. Nucleus. 2026;17(1):2690847. doi: 10.1080/19491034.2026.2690847 42411057 PMC13348924

[83] Jung-Garcia Y, Maiques O, Monger J, et al. LAP1 supports nuclear adaptability during constrained melanoma cell migration and invasion. Nat Cell Biol. 2023;25(1):108–119. doi: 10.1038/s41556-022-01042-3 36624187 PMC9859759

[84] Caravia XM, Ramirez-Martinez A, Gan P, et al. Loss of function of the nuclear envelope protein LEMD2 causes DNA damage-dependent cardiomyopathy. J Clin Invest. 2022;132(22):e158897. doi: 10.1172/JCI158897 36377660 PMC9663152

[85] Lammerding J, Fong LG, Ji JY, et al. Lamins A and C but not lamin B1 regulate nuclear mechanics. J Biol Chem. 2006;281(35):25768–25780. doi: 10.1074/jbc.M513511200 16825190

[86] Lammerding J, Hsiao J, Schulze PC, et al. Abnormal nuclear shape and impaired mechanotransduction in emerin-deficient cells. J Cell Biol. 2005;170(5):781–791. doi: 10.1083/jcb.200502148 16115958 PMC2171355

[87] Rowat AC, Lammerding J, Ipsen JH. mechanical properties of the Cell nucleus and the effect of emerin deficiency. Biophys J. 2006;91(12):4649–4664. doi: 10.1529/biophysj.106.086454 16997877 PMC1779937

[88] Chen NY, Kim PH, Tu Y, et al. Increased expression of LAP2β eliminates nuclear membrane ruptures in nuclear lamin-deficient neurons and fibroblasts. Proc Natl Acad Sci USA. 2021;118(25):e2107770118. doi: 10.1073/pnas.2107770118 34161290 PMC8237679

[89] Alabi Y, Aksenova V, Arnaoutov A, et al. Lamin B1 and LAP2β resist cytoskeletal force to maintain lamin A/C meshwork organization and preserve nuclear integrity. Mol Biol Cell. 2025;36(9):ar111. doi: 10.1091/mbc.E25-02-0057 40668606 PMC12415607

[90] Ross JA, Arcos-Villacis N, Battey E, et al. Lem2 is essential for cardiac development by maintaining nuclear integrity. Cardiovasc Res. 2023;119(11):2074–2088. doi: 10.1093/cvr/cvad061 37067297 PMC10478753

[91] Stephens AD, Liu PZ, Banigan EJ, et al. Chromatin histone modifications and rigidity affect nuclear morphology independent of lamins. Mol Biol Cell. 2018;29(2):220–233. doi: 10.1091/mbc.E17-06-0410 29142071 PMC5909933

[92] Nmezi B, Xu J, Fu R, et al. Concentric organization of A- and B-type lamins predicts their distinct roles in the spatial organization and stability of the nuclear lamina. Proc Natl Acad Sci USA. 2019;116(10):4307–4315. doi: 10.1073/pnas.1810070116 30765529 PMC6410836

[93] Xie W, Chojnowski A, Boudier T, et al. A-type lamins form distinct filamentous networks with differential nuclear pore complex associations. Curr Biol. 2016;26(19):2651–2658. doi: 10.1016/j.cub.2016.07.049 27641764

[94] Shimi T, Kittisopikul M, Tran J, et al. Structural organization of nuclear lamins A, C, B1, and B2 revealed by superresolution microscopy. Mol Biol Cell. 2015;26(22):4075–4086. doi: 10.1091/mbc.E15-07-0461 26310440 PMC4710238

[95] Negri ML, D’Annunzio S, Vitali G, et al. May the force be with you: nuclear condensates function beyond transcription control: potential nongenetic functions of nuclear condensates in physiological and pathological conditions. Bioessays. 2023;45(10):e2300075. doi: 10.1002/bies.202300075 37530178

[96] Zhao JZ, Xia J, Brangwynne CP. mechanical regulation and activity of nuclear condensates. Biophys J. 2025;S0006-3495(25):00660–5. doi: 10.1016/j.bpj.2025.10.00941063457

[97] McCreery KP, Stubb A, Stephens R, et al. Mechano-osmotic signals control chromatin state and fate transitions in pluripotent stem cells. Nat Cell Biol. 2025;27(10):1757–1770. doi: 10.1038/s41556-025-01767-x 41023488 PMC12527910

[98] Elpers MA, Odell JD, Shu T, et al. Polarization increases nuclear stiffness in macrophages despite reduction in lamin A/C levels. 2025. doi: 10.1101/2025.11.22.689940 PMC13229938 42245196

[99] Ding X, Hao H, Elnatan D, et al. Giant KASH proteins and ribosomes establish distinct cytoplasmic biophysical properties in vivo. Sci Adv. 2025;11(37):eadx0952. doi: 10.1126/sciadv.adx0952 40929259 PMC12422203

[100] Elpers MA, Odell J, Henretta SJ, et al. Polarization increases nuclear stiffness in macrophages despite reduction in lamin A/C levels. NPJ Biol Phys Mech. 2026;3(1):8. doi: 10.1038/s44341-026-00038-6 42245196 PMC13229938

[101] Roberts AB, Zhang J, Raj Singh V, et al. Tumor cell nuclei soften during transendothelial migration. J Biomech. 2021;121:110400. doi: 10.1016/j.jbiomech.2021.110400 33882444 PMC8274349

[102] Chalut KJ, Höpfler M, Lautenschläger F, et al. Chromatin decondensation and nuclear softening accompany Nanog downregulation in embryonic stem cells. Biophys J. 2012;103(10):2060–2070. doi: 10.1016/j.bpj.2012.10.015 23200040 PMC3512036

[103] Heo SJ, Driscoll TP, Thorpe SD, et al. Differentiation alters stem cell nuclear architecture, mechanics, and mechano-sensitivity. Elife. 2016;5:e18207. doi: 10.7554/eLife.18207 27901466 PMC5148611

[104] Ihalainen TO, Aires L, Herzog FA, et al. Differential basal-to-apical accessibility of lamin A/C epitopes in the nuclear lamina regulated by changes in cytoskeletal tension. Nat Mater. 2015;14(12):1252–1261. doi: 10.1038/nmat4389 26301768 PMC4655446

[105] Buxboim A, Swift J, Irianto J, et al. Matrix elasticity regulates lamin-A,C phosphorylation and turnover with feedback to Actomyosin. Curr Biol. 2014;24(16):1909–1917. doi: 10.1016/j.cub.2014.07.001 25127216 PMC4373646

[106] Shen Z, Gelashvili Z, Niethammer P. Endoplasmic reticulum disruption stimulates nuclear membrane mechanotransduction. Nat Cell Biol. 2026;28(1):125–134. doi: 10.1038/s41556-025-01820-9 41366519 PMC12807876

[107] Booth-Gauthier EA, Alcoser TA, Yang G, et al. Force-induced changes in subnuclear movement and rheology. Biophys J. 2012;103(12):2423–2431. doi: 10.1016/j.bpj.2012.10.039 23260044 PMC3525843

[108] Schuller AP, Wojtynek M, Mankus D, et al. The cellular environment shapes the nuclear pore complex architecture. Nature. 2021;598(7882):667–671. doi: 10.1038/s41586-021-03985-3 34646014 PMC8550940

[109] Wing CE, Fung HYJ, Chook YM. Karyopherin-mediated nucleocytoplasmic transport. Nat Rev Mol Cell Biol. 2022;23(5):307–328. doi: 10.1038/s41580-021-00446-7 35058649 PMC10101760

[110] Kofler M, Kapus A. Nuclear import and export of YAP and TAZ. Cancers (Basel). 2023;15(20):4956. doi: 10.3390/cancers15204956 37894323 PMC10605228

[111] Khatau SB, Hale CM, Stewart-Hutchinson PJ, et al. A perinuclear actin cap regulates nuclear shape. Proc Natl Acad Sci USA. 2009;106(45):19017–19022. doi: 10.1073/pnas.0908686106 19850871 PMC2776434

[112] Chancellor TJ, Lee J, Thodeti CK, et al. Actomyosin tension exerted on the nucleus through Nesprin-1 connections influences endothelial Cell adhesion, migration, and cyclic strain-induced reorientation. Biophys J. 2010;99(1):115–123. doi: 10.1016/j.bpj.2010.04.011 20655839 PMC2895377

[113] Neumann S, Schneider M, Daugherty RL, et al. Nesprin-2 interacts with α-catenin and regulates wnt signaling at the nuclear envelope. J Biol Chem. 2010;285(45):34932–34938. doi: 10.1074/jbc.M110.119651 20801886 PMC2966107

[114] Lombardi ML, Jaalouk DE, Shanahan CM, et al. The interaction between nesprins and sun proteins at the nuclear envelope is critical for force transmission between the nucleus and cytoskeleton. J Biol Chem. 2011;286(30):26743–26753. doi: 10.1074/jbc.M111.233700 21652697 PMC3143636

[115] Morgan JT, Pfeiffer ER, Thirkill TL, et al. Nesprin-3 regulates endothelial cell morphology, perinuclear cytoskeletal architecture, and flow-induced polarization. Mol Biol Cell. 2011;22(22):4324–4334. doi: 10.1091/mbc.E11-04-0287 21937718 PMC3216658

[116] Stewart RM, Zubek AE, Rosowski KA, et al. Nuclear–cytoskeletal linkages facilitate cross talk between the nucleus and intercellular adhesions. J Cell Bio. 2015;209(3):403–418. doi: 10.1083/jcb.201502024 25963820 PMC4427780

[117] Shen KM, Shields EJ, Barka V, et al. The cytoskeleton contributes to abnormal genome-lamina interactions in LMNA-deficient cardiomyocytes. J Cell Biol. 2026;225(5):e202506137. doi: 10.1083/jcb.202506137 41891953 PMC13040498

[118] Lee YL, Burke B. LINC complexes and nuclear positioning. Semin Cell Dev Biol. 2018;82:67–76. doi: 10.1016/j.semcdb.2017.11.008 29191370

[119] Sosa BA, Rothballer A, Kutay U, et al. LINC complexes form by binding of Three KASH peptides to domain interfaces of trimeric SUN proteins. Cell. 2012;149(5):1035–1047. doi: 10.1016/j.cell.2012.03.046 22632968 PMC3383001

[120] Zhou Z, Du X, Cai Z, et al. Structure of Sad1-UNC84 homology (SUN) domain defines features of molecular bridge in nuclear envelope. J Biol Chem. 2012;287(8):5317–5326. doi: 10.1074/jbc.M111.304543 22170055 PMC3285312

[121] Rothballer A, Schwartz TU, Kutay U. Lincing complex functions at the nuclear envelope: what the molecular architecture of the LINC complex can reveal about its function. Nucleus. 2013;4(1):29–36. doi: 10.4161/nucl.23387 23324460 PMC3585024

[122] Cruz VE, Esra Demircioglu F, Schwartz TU. Structural analysis of different LINC complexes reveals distinct binding modes. J Mol Biol. 2020;432(23):6028–6041. doi: 10.1016/j.jmb.2020.09.019 33058875 PMC11552096

[123] Lim SM, Cruz VE, Antoku S, et al. Structures of FHOD1-Nesprin1/2 complexes reveal alternate binding modes for the FH3 domain of formins. Structure. 2021;29(6):540–552.e5. doi: 10.1016/j.str.2020.12.013 33472039 PMC9122090

[124] Jahed Z, Shams H, Mofrad MRK. A disulfide bond is required for the transmission of forces through SUN-KASH complexes. Biophys J. 2015;109(3):501–509. doi: 10.1016/j.bpj.2015.06.057 26244732 PMC4571000

[125] Cain NE, Jahed Z, Schoenhofen A, et al. Conserved SUN-KASH interfaces mediate LINC complex-dependent nuclear movement and positioning. Curr Biol. 2018;28(19):3086–3097.e4. doi: 10.1016/j.cub.2018.08.001 30245107 PMC6219386

[126] Gurusaran M, Davies OR. A molecular mechanism for LINC complex branching by structurally diverse SUN-KASH 6: 6 assemblies. Elife. 2021;10:e60175. doi: 10.7554/eLife.60175 33393904 PMC7800377

[127] Hennen J, Saunders CA, Mueller JD, et al. Fluorescence fluctuation spectroscopy reveals differential SUN protein oligomerization in living cells. Mol Biol Cell. 2018;29(9):1003–1011. doi: 10.1091/mbc.E17-04-0233 29514929 PMC5921568

[128] King MC. Dynamic regulation of LINC complex composition and function across tissues and contexts. FEBS Lett. 2023;597(22):2823–2832. doi: 10.1002/1873-3468.14757 37846646

[129] Kanchanawong P, Shtengel G, Pasapera AM, et al. Nanoscale architecture of integrin-based cell adhesions. Nature. 2010;468(7323):580–584. doi: 10.1038/nature09621 21107430 PMC3046339

[130] Kanchanawong P, Calderwood DA. Organization, dynamics and mechanoregulation of integrin-mediated cell-ECM adhesions. Nat Rev Mol Cell Biol. 2023;24(2):142–161. doi: 10.1038/s41580-022-00531-5 36168065 PMC9892292

[131] Zaidel-Bar R, Itzkovitz S, Ma’ayan A, et al. Functional atlas of the integrin adhesome. Nat Cell Biol. 2007;9(8):858–867. doi: 10.1038/ncb0807-858 17671451 PMC2735470

[132] Horton ER, Byron A, Askari JA, et al. Definition of a consensus integrin adhesome and its dynamics during adhesion complex assembly and disassembly. Nat Cell Biol. 2015;17(12):1577–1587. doi: 10.1038/ncb3257 26479319 PMC4663675

[133] Patla I, Volberg T, Elad N, et al. Dissecting the molecular architecture of integrin adhesion sites by cryo-electron tomography. Nat Cell Biol. 2010;12(9):909–915. doi: 10.1038/ncb2095 20694000

[134] Bennett V, Lorenzo DN. Spectrin- and ankyrin-based membrane domains and the evolution of vertebrates. Curr Top Membr. 2013;72:1–37. doi: 10.1016/B978-0-12-417027-8.00001-5 24210426

[135] Machnicka B, Czogalla A, Hryniewicz-Jankowska A, et al. Spectrins: a structural platform for stabilization and activation of membrane channels, receptors and transporters. Biochim Biophys Acta. 2014;1838(2):620–634. doi: 10.1016/j.bbamem.2013.05.002 23673272

[136] Mislow JMK, Holaska JM, Kim MS, et al. Nesprin-1alpha self-associates and binds directly to emerin and lamin a in vitro. FEBS Lett. 2002;525(1–3):135–140. doi: 10.1016/s0014-5793(02)03105-8 12163176

[137] Lu W, Schneider M, Neumann S, et al. Nesprin interchain associations control nuclear size. Cell Mol Life Sci. 2012;69(20):3493–3509. doi: 10.1007/s00018-012-1034-1 22653047 PMC11114684

[138] Rajgor D, Shanahan CM. Nesprins: from the nuclear envelope and beyond. Expert Rev Mol Med. 2013;15:e5. doi: 10.1017/erm.2013.6 23830188 PMC3733404

[139] Kutscheidt S, Zhu R, Antoku S, et al. FHOD1 interaction with nesprin-2G mediates TAN line formation and nuclear movement. Nat Cell Biol. 2014;16(7):708–715. doi: 10.1038/ncb2981 24880667 PMC4113092

[140] Taranum S, Sur I, Müller R, et al. Cytoskeletal interactions at the nuclear envelope mediated by nesprins. Int J Cell Biol. 2012;2012:1–11. doi: 10.1155/2012/736524 PMC3296292 22518138

[141] Ding X, Kumari S, Gregory E, et al. Muscular dystrophy-associated lamin variants disrupt cellular organization through a nucleolar-ribosomal axis controlling cytoplasmic macromolecular crowding. bioRxiv: Prepr Serv Biol. 2025. doi: 10.1101/2025.09.22.677926

[142] Zhang Q, Ragnauth CD, Skepper JN, et al. Nesprin-2 is a multi-isomeric protein that binds lamin and emerin at the nuclear envelope and forms a subcellular network in skeletal muscle. J Cell Sci. 2005;118(Pt 4):673–687. doi: 10.1242/jcs.01642 15671068

[143] Guo Y, Zheng Y. Lamins position the nuclear pores and centrosomes by modulating dynein. Mol Biol Cell. 2015;26(19):3379–3389. doi: 10.1091/mbc.E15-07-0482 26246603 PMC4591684

[144] Goldberg MW. Nuclear pore complex tethers to the cytoskeleton. Semin Cell Dev Biol. 2017;68:52–58. doi: 10.1016/j.semcdb.2017.06.017 28676424

[145] Salpingidou G, Smertenko A, Hausmanowa-Petrucewicz I, et al. A novel role for the nuclear membrane protein emerin in association of the centrosome to the outer nuclear membrane. J Cell Biol. 2007;178(6):897–904. doi: 10.1083/jcb.200702026 17785515 PMC2064615

[146] Holaska JM, Wilson KL. An emerin “proteome”: purification of distinct emerin-containing complexes from HeLa cells suggests molecular basis for diverse roles including gene regulation, mRNA splicing, signaling, mechanosensing, and nuclear architecture. Biochemistry. 2007;46(30):8897–8908. doi: 10.1021/bi602636m 17620012 PMC2635128

[147] Ganguly A, Zmuda H, Abello J, et al. The Nemp1–Nesprin complex mediates cellular responses to matrix mechanics. Proc Natl Acad Sci. 2026;123(9):e2521253123. doi: 10.1073/pnas.2521253123 41730104 PMC12956887

[148] Irianto J, Pfeifer CR, Ivanovska IL, et al. Nuclear lamins in cancer. Cell Mol Bioeng. 2016;9(2):258–267. doi: 10.1007/s12195-016-0437-8 27570565 PMC4999255

[149] Zuela-Sopilniak N, Lammerding J. Can’t handle the stress? Mechanobiology and disease. Trends Mol Med. 2022;28(9):710–725. doi: 10.1016/j.molmed.2022.05.010 35717527 PMC9420767

[150] Henretta S, Lammerding J. Nuclear envelope proteins, mechanotransduction, and their contribution to breast cancer progression. NPJ Biol Phys Mech. 2025;2(1):14. doi: 10.1038/s44341-025-00018-2 40337116 PMC12052594

[151] Greiner AM, Klein F, Gudzenko T, et al. Cell type-specific adaptation of cellular and nuclear volume in micro-engineered 3D environments. Biomaterials. 2015;69:121–132. doi: 10.1016/j.biomaterials.2015.08.016 26283159

[152] Jain N, Vogel V. Spatial confinement downsizes the inflammatory response of macrophages. Nat Mater. 2018;17(12):1134–1144. doi: 10.1038/s41563-018-0190-6 30349032 PMC6615903

[153] Wilson RE, Denisin AK, Dunn AR, et al. 3D microwell platforms for control of single Cell 3D geometry and intracellular organization. Cell Mol Bioeng. 2021;14(1):1–14. doi: 10.1007/s12195-020-00646-9 33643464 PMC7878614

[154] Remuzzi A, Bonandrini B, Tironi M, et al. Effect of the 3D artificial Nichoid on the morphology and mechanobiological response of mesenchymal stem cells cultured in vitro. Cells. 2020;9(8):1873. doi: 10.3390/cells9081873 32796521 PMC7464958

[155] Kalukula Y, Ciccone G, Mohammed D, et al. Unlocking the therapeutic potential of cellular mechanobiology. Sci Adv. 2025;11(44):eaea6817. doi: 10.1126/sciadv.aea6817 41171907 PMC12577691

[156] Smith ER, Wang JQ, Yang DH, et al. Paclitaxel resistance related to nuclear envelope structural sturdiness. Drug Resist Updat. 2022;65:100881. doi: 10.1016/j.drup.2022.100881 36368286

[157] Hale T, Hale VL, Kolata P, et al. Paclitaxel compromises nuclear integrity in interphase through SUN2-mediated cytoskeletal coupling. J Cell Sci. 2026;139(12):jcs264494. doi: 10.1242/jcs.264494 41367359 PMC12863304

